# Inter-site comparability of 4D flow cardiovascular magnetic resonance measurements in healthy traveling volunteers—a multi-site and multi-magnetic field strength study

**DOI:** 10.3389/fcvm.2024.1456814

**Published:** 2024-11-08

**Authors:** Maximilian Müller, Elias Daud, Georg Langer, Jan Gröschel, Darian Viezzer, Thomas Hadler, Ning Jin, Daniel Giese, Sebastian Schmitter, Jeanette Schulz-Menger, Ralf F. Trauzeddel

**Affiliations:** ^1^ Corporate Member of Freie Universität Berlin and Humboldt-Universität zu Berlin, ECRC Experimental and Clinical Research Center, Charité – Universitätsmedizin Berlin, Berlin, Germany; ^2^ Working Group on Cardiovascular Magnetic Resonance, Experimental and Clinical Research Center, A Joint Cooperation Between the Charité–Universitätsmedizin Berlin and the Max-Delbrück-Center for Molecular Medicine, Berlin, Germany; ^3^ DZHK (German Center for Cardiovascular Research), Berlin, Germany; ^4^ The Cardiology Department, Galilee Medical Center, Azrieli Faculty of Medicine Bar-Ilan University, Nahariya - Safed, Israel; ^5^ Klinik für Kardiologie, Angiologie und Intensivmedizin, Deutsches Herzzentrum der Charité – Medical Heart Center of Charité and German Heart Institute Berlin, Berlin, Germany; ^6^ Cardiovascular MR R&D, Siemens Medical Solutions USA, Inc., Cleveland, OH, United States; ^7^ Cardiovascular MR R&D, Siemens Healthcare GmbH, Erlangen, Germany; ^8^ Institute of Radiology, University Hospital Erlangen, Friedrich-Alexander-Universität Erlangen-Nürnberg (FAU), Erlangen, Germany; ^9^ Physikalisch-Technische Bundesanstalt (PTB), Braunschweig/Berlin, Germany; ^10^ Center for Magnetic Resonance Research, University of Minnesota, Minneapolis, MN, United States; ^11^ Medical Physics in Radiology, German Cancer Research Center (DKFZ), Heidelberg, Germany; ^12^ Department of Cardiology and Nephrology, HELIOS Klinikum Berlin Buch, Berlin, Germany; ^13^ Department of Anesthesiology and Intensive Care Medicine, Charité Campus Benjamin Franklin, Charité - Universitätsmedizin Berlin, Corporate Member of Freie Universität Berlin and Humboldt-Universität zu Berlin, Berlin, Germany

**Keywords:** 4D flow CMR, healthy volunteers, thoracic aorta, standardization, quality assurance

## Abstract

**Background:**

Time-resolved 3D cine phase-contrast cardiovascular magnetic resonance (4D flow CMR) enables the characterization of blood flow using basic and advanced hemodynamic parameters. However, different confounders, e.g., different field strength, scanner configurations, or sequences, might impact 4D flow CMR measurements. This study aimed to analyze the inter-site reproducibility of 4D flow CMR to determine the influence of said confounders.

**Methods:**

A cohort of 19 healthy traveling volunteers underwent 4D flow CMR at four different sites (Sites I–III: 3 T scanner; Site IV: 1.5 T scanner; all Siemens Healthineers, Erlangen, Germany). Two protocols of one 4D flow CMR research sequence were performed, one acquiring velocity vector fields in the thoracic aorta only and one in the entire heart and thoracic aorta combined. Basic and advanced hemodynamic parameters, i.e., forward flow volume (FFV), peak and mean velocities (Vp and Vm), and wall shear stress (3D WSS), at nine different planes across the thoracic aorta (P1–P2 ascending aorta, P3–P5 aortic arch, P6–P9 descending aorta) were analyzed. Based on a second scan at Site I, mean values and tolerance ranges (TOL) were generated for inter-site comparison. Equivalency was assumed when confidence intervals of Sites II–IV lay within such TOL. Additionally, inter- and intra-observer analysis as well as a comparison between the two protocols was performed, using an intraclass correlation coefficient (ICC).

**Results:**

Inter-site comparability showed equivalency in P1 and P2 for FFV, Vp, and Vm at all sites. Non-equivalency was present in various planes of P3–P9 and in P2 for 3D WSS in one protocol. In total, Site IV showed the most disagreements. Protocol comparison yielded excellent (>0.9) ICC in every plane for FFV, good (0.75–0.9) to excellent ICC for Vm and 3D WSS, good to excellent ICC in eight planes for Vp, and moderate (0.5–0.75) ICC in one plane for Vp. Inter- and intra-observer analysis showed excellent agreement for every parameter.

**Conclusions:**

Basic and advanced hemodynamic parameters revealed equivalency at different sites and field strength in the ascending aorta, a clinically important region of interest, under a highly controlled environment.

## 1 Introduction

Cardiovascular magnetic resonance imaging (CMR) plays an important role in clinical routine as it presents the gold standard of non-invasive cardiac function analysis and myocardial tissue characterization. Beyond that, three-dimensional cine phase-contrast cardiovascular magnetic resonance with three-directional velocity encoding (4D flow CMR) enables the comprehensive assessment of hemodynamics in CMR (1, 2). With its potential to display the blood velocity vector field temporally and 3D spatially resolved and to examine advanced blood flow parameters, e.g., wall shear stress (WSS) and kinetic energies, it could serve as an additional diagnostic tool and risk stratification instrument in various cardiovascular diseases such as aortic dilation and aneurysms as well as aortic dissections (3–8) in addition to the currently in clinically routine used, namely, two-dimensional (2D) flow CMR (9, 10). Additionally, some 4D flow CMR sequences provide the possibility to not only examine hemodynamics in large blood vessels but also display and analyze intraventricular and transvalvular flow (2, 11). However, to cover all anatomical structures of interest, i.e., the heart with its chambers and valves as well as the intrathoracic great vessels, there is a necessity to increase the field of view. Therefore, larger distances from the isocenter resulting in possible offset errors have to be considered when applying such sequences (2).

Since its introduction, 4D flow CMR has become an important tool for the diagnosis and monitoring of patients with congenital heart diseases (12).

However, even though 4D flow CMR was introduced more than two decades ago, in the field of adult cardiology, it is still mainly used in research. This may be due to several reasons. Besides the limited comparability of different postprocessing software solutions, it has been shown that 4D flow CMR-derived hemodynamic parameters from different vendors and different field strengths show significant differences (13–15). Furthermore, the detailed implementation of the 4D flow CMR sequences and certain parameters such as the encoding scheme or the echo time can have an impact on derived parameters and such details often vary between different vendors (16). So far, however, it has not been analyzed whether this applies to 4D flow CMR images acquired at different scanners from the same vendor.

Therefore, this study aimed to (1) evaluate whether different MR scanners of the same vendor at different sites show comparable quantitative results based on 4D flow CMR and to reduce the number of possible confounders and (2) investigate if two protocols of one 4D flow CMR research sequence with different fields of view yield good agreement for basic and advanced hemodynamic parameters in the thoracic aorta.

## 2 Materials and methods

### 2.1 Study design and population

**Figure 1:**
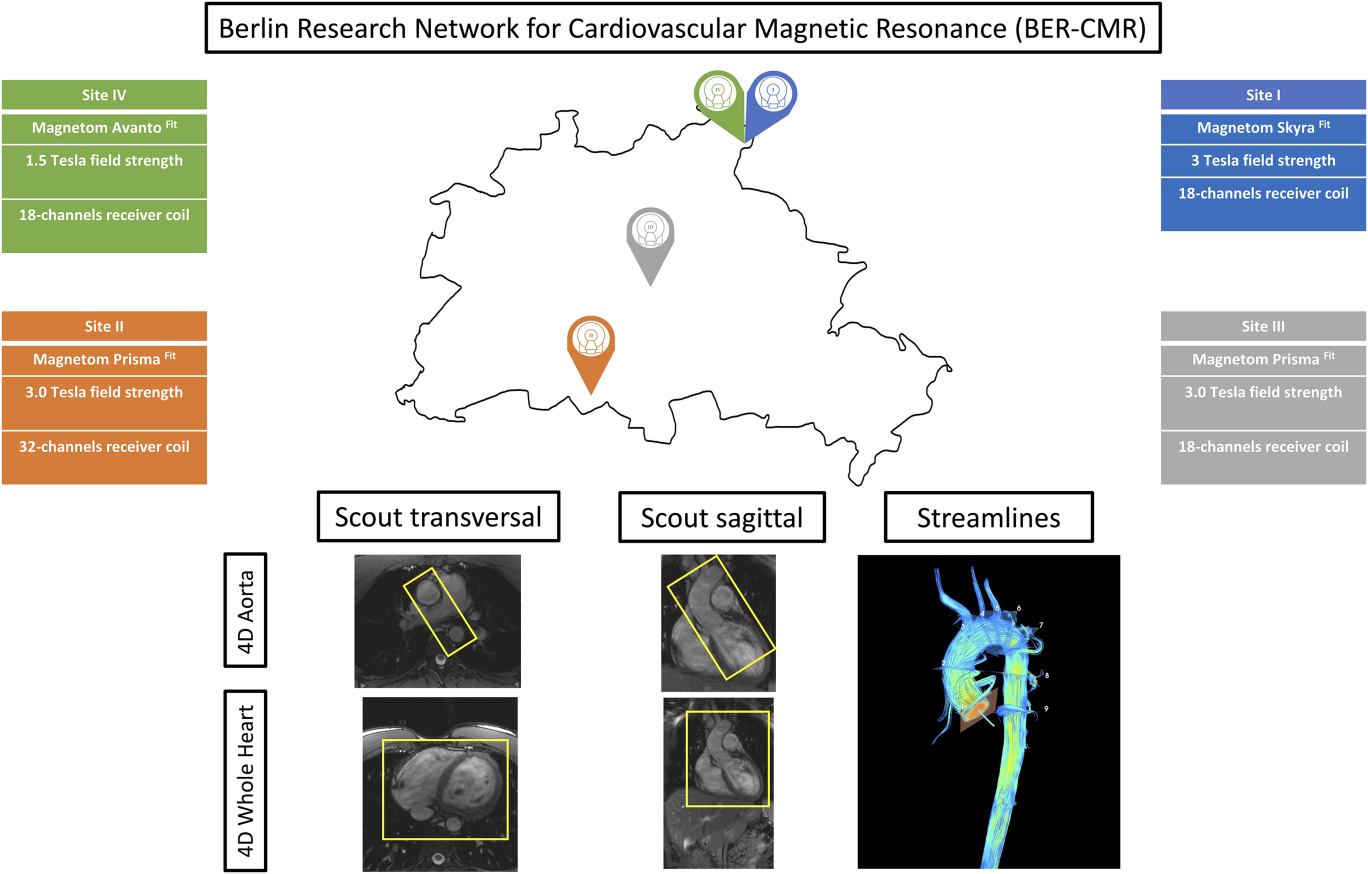
Berlin Research Network for CMR (BER-CMR). Each colored pin demonstrates one study site. Information about scanner type, field strength, and coils used are given and color-coded in the respective site color. The sagittal and transversal scout images display the fields of view of the aorta and whole heart protocol separately. Streamlines visualize the results of both protocols in use in one image.

A prospective observational study was conducted across the different sites of the Berlin Research Network for CMR (BER-CMR), an association of different sites across the Charité – Universitätsmedizin Berlin applying CMR for clinical and scientific purposes (Figure 1) (17). The study was approved by the local ethics committee at Charité – Universitätsmedizin Berlin (EA1/183/19) and conducted in accordance with the Declaration of Helsinki and its later amendments. Informed written consent was prospectively obtained from all study participants. The study was registered at ISRCTN (ISRCTN 14627679) and funded by the German Center for Cardiovascular Research (DZHK) (funding number 81Z0100208). Twenty healthy volunteers were screened for eligibility and recruited over a period of 1 year and 9 months, from August 2020 to April 2022. The inclusion criteria were age ≥18 years, given consent to report incidental findings that might be seen during CMR scans, no known prior cardiac or vascular diseases, no history of oral medication intake for cardiovascular treatment, and no pathologies present on a 12-lead electrocardiogram (ECG). The exclusion criteria were a lack of ability to consent to study participation, contraindications against the performance of a CMR study, and pregnancy or breastfeeding at the time of study participation.

### 2.2 Image acquisition

Five CMR scans at the four different sites of the BER-CMR were planned for each participant. The BER-CMR included three 3 T scanners and one 1.5 T system, all from the same manufacturer: Site I, MAGNETOM Skyra fit 3 T; Sites II and III, MAGNETOM Prisma fit 3 T; and Site IV, MAGNETOM Avanto fit 1.5 T (Siemens Healthineers, Erlangen, Germany). At Site I, the full scan was repeated after a 10–20 min break. During this break, the individual study participant was taken off the scanner and repositioned after the break. This step made it necessary to reposition the coil and replan image acquisition. The scan protocol and sequence parameters were harmonized across all scanners beforehand, except for adaptions necessary due to field strengths and scanner hardware configuration heterogeneities. For 4D flow CMR acquisition, two different protocols using a 4D flow CMR research sequence (Siemens Healthineers, Erlangen, Germany) were implemented at each site, one for acquiring 4D flow magnetic resonance imaging (MRI) data in the thoracic aorta alone (aorta) and another for acquiring 4D flow MRI data in the entire heart and thoracic aorta combined (whole heart). Both protocols relied on compressed sensing with an acceleration factor of 7.6. The first was acquired using a sagittal oblique volume, controlled in a transversal view, covering the entire thoracic aorta. For the second, a rectangular volume was placed transversally and controlled in a sagittal view, over the entire heart and intrathoracic great vessels (Figure 1). Retrospective ECG gating in combination with a respiratory navigator, placed on the lung–liver interface, was used. Scan parameters for both protocols are provided in Table 1. Prior to the acquisition of 4D flow images, a 2D flow CMR scout covering the ascending aorta at the sinotubular junction was performed to define the velocity encoding (V_enc_), testing for 150, 200, and 250 cm/s (18). In the absence of aliasing, 150 cm/s was chosen; otherwise, the next higher velocity was chosen, as proposed in the literature and the recent consensus statement on 4D flow CMR (2, 19). The number of slices, distance factor, and velocity encoding sensitivity (V_enc_) in both 4D flow CMR protocols have been adapted in the acquisition of the second to last scan according to the settings in the first scan. This was done for each traveling volunteer individually. For cardiac chamber and left ventricular function quantification, standard steady-state free-precision cine images were acquired.

**Table 1:** Sequence parameters.

Parameter	4D flow CMR aorta	4D flow CMR whole heart
Phase encoding direction	A >> P	A >> P
Distance factor (percent)	20	20
Slice thickness (mm)	2.30	2.30
Repetition time (ms)	41.12	40.80
Echo time (ms)	2.44	2.4
Velocity encoding (cm/s)	150–200	150–200
Flip angle (degrees)	8	8
Voxel size (mm × mm)	2.3 × 2.3 × 2.3	2.3 × 2.3 × 2.3
Calculated phases	20	20
Bandwidth (Hz/Px)	460	460
Acceleration rate	7.6	7.6
Acceleration technique	Compressed sensing	Compressed sensing

A, anterior; P, posterior.

### 2.3 Image postprocessing

**Figure 2:**
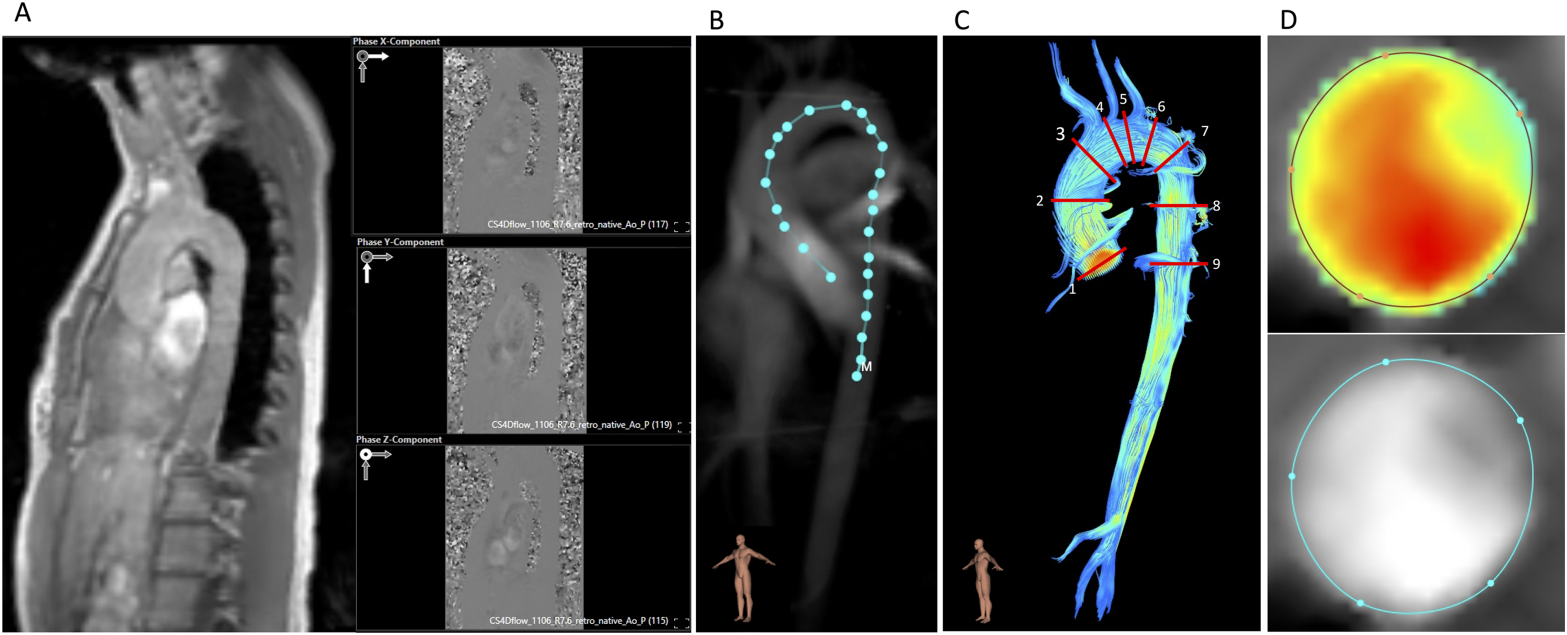
Postprocessing of 4D flow CMR. **(A)** Magnitude image and the three affiliated phase encoding images (x, y, z). **(B)** Vessel centerline placement along the thoracic aorta. **(C)** Positions of the nine analysis planes. **(D)** Velocity masks and magnitude images for correction of lumen contour segmentation in every cardiac phase.

Postprocessing of 4D flow CMR was performed by a single observer (MM, with 1.5 years of experience in 4D flow CMR) using Caas MR Solutions 5.2 (Caas, Pie Medical Imaging BV, Maastricht, The Netherlands). Aorta and whole heart datasets were postprocessed using the same software tool of the mentioned software. After choosing the magnitude image, three affiliated phase encoding images were selected automatically by the program and matched with the x-, y-, and z-directions. The selection was reviewed and if necessary corrected by the observer (Figure 2A). Aliasing and background phase correction were done as previously described in the literature (13). Afterwards, a centerline was extracted along the thoracic aorta with the starting point placed in the left ventricular outflow tract (LVOT) and the end point in the abdominal aorta right below the diaphragm (Figure 2B). The starting point, the end point, and reference points along the thoracic aorta were placed manually, and delineation of the centerline was then performed fully automatically. Based on the centerline and phase encoding images, a 3D image of the thoracic aorta is generated and can be used for anatomical reference. In the following step, nine planes (P) were placed along the thoracic aorta at pre-defined locations: (1) sinotubular junction; (2) mid-ascending aorta at the height of the pulmonary bifurcation; (3) proximal to the origin of the brachiocephalic trunk; (4) distal of the origin of the brachiocephalic trunk; (5) between the left common carotid artery and the left subclavian artery; (6) distal the left subclavian artery; (7) aortic isthmus; (8) descending aorta at the same level as plane 2; and (9) descending aorta at the same level as plane 1 (Figure 2C). Each plane is placed perpendicular to the vessel wall and centerline by the postprocessing software itself. Afterwards, regions of interest (ROI) in every plane for every phase were defined based on magnitude images as well as velocity maps to define the vessel wall boundaries (Figure 2D) (20).

For every plane, basic and advanced hemodynamic parameters, i.e., forward flow volume (FFV), peak systolic and mean velocities (Vp and Vm, respectively), and wall shear stress (3D WSS), were extracted. 3D WSS is provided in 90 segments over the whole aortic circumference in each plane and phase resulting in 1,800 segments per plane by Caas MR Solutions. We defined 3D WSS as the average of the values at the peak systolic phase and the adjacent ± two phases as previously described (14).

### 2.4 Statistical analysis

Based on the intra-scanner and inter-scanner at Site I, the mean values and their 95% tolerance ranges (TOL) were calculated for each parameter. These TOL were used to assess the acceptability of differences for inter-site comparisons. The remaining three sites were then compared to this reference site by calculating the mean deviation with a 95% confidence interval (95% CI). If the CI was contained within the TOL, equivalency was assumed as previously published (21). Besides inter-site comparison, the comparability of 4D flow CMR aorta and whole heart hemodynamic results was examined. Based on the corresponding values of each scan, the mean values and mean deviations were generated per plane and site. Additionally, the mean values ± standard deviation were calculated for each parameter per plane and per protocol. For inter-observer analysis, a second reader with 6 years of cumulative experience in 4D flow CMR (RT) repeated analyses of the measurements before repositioning at Site I in 10 randomly selected traveling volunteers. This was also done for intra-observer analysis. Intraclass correlation coefficients (ICC) with a two-way mixed model and absolute agreement with 95% CI as well as the mean difference with the 95% limits of agreement displayed using Bland–Altman plots were calculated for the inter- and intra-observer analysis. For protocol comparability, ICC values with 95% CI and mean values ± 95% limits of agreement have been calculated as well. ICC was interpreted as follows: >0.9 excellent, >0.75 good, >0.5 moderate, else poor (22). Statistical analysis was done using SAS 9.4 (SAS Institute Inc., Cary, NC, USA) and GraphPad Prism version 9 (GraphPad Software, San Diego, CA, USA).

## 3 Results

### 3.1 Basic characteristics of the study cohort

Nineteen traveling volunteers (11 males/8 females) could be included in the final analysis as one study participant had to be excluded due to a newly diagnosed cardiovascular disease timely after the scans. From the remaining 19 volunteers, 1 could not be scanned at Site I, 2 could not be scanned at Site II due to issues with the license of the sequence, and 1 could not be scanned at Site III due to a copper spiral *in situ* and the special rules of this scanner, which is dedicated to research only while being a certified “product scanner.” Three 4D flow CMR aorta and six whole heart measurements could not be analyzed due to postprocessing issues, e.g., the postprocessing tool was unable to open the magnitude and phase-contrast images in the respective window or insufficient image quality, e.g., the postprocessing tool was unable to generate a centerline. In total, 87 4D flow CMR aorta and 84 4D flow CMR whole heart measurements, 95% of the conducted scans, were included in the statistical analysis. The average scan duration, from the beginning of each measurement until the end of reconstruction, was 7.89 ± 2.00 min for the aorta protocol and 16.71 ± 5.10 min for the whole heart protocol. The median time interval between the first and the last scan for one individual traveling volunteer was 8 days with an interquartile range of 48 days.

Aliasing was present in no scan that was conducted throughout this study.

Baseline characteristics are displayed in Table 2. The 19 traveling volunteers had the following absolute and standardized body surface area and height parameters for left and right ventricles (LV and RV, respectively): LV end-diastolic volume = 181 ± 42 mL, 96 ± 17 mL/m^2^, 100 ± 20 mL/m; LV stroke volume = 113 ± 27 mL, 60 ± 17 mL/m^2^; LV ejection fraction = 62% ± 3%; RV end-diastolic volume = 204 ± 51 mL, 108 ± 21 mL/m^2^; RV stroke volume = 107 ± 27 mL, 56 ± 11 mL/m^2^; and RV ejection fraction = 52% ± 4%.

**Table 2:** Baseline characteristics.

Parameter	Site I	Site II	Site III	Site IV
N	18	17	18	19
Female/male	8/10	7/10	7/11	8/11
Age (years)	26.3 ± 6.1	24.6 ± 3.5	26.3 ± 6.2	26.1 ± 6
Height (m)	1.8 ± 0.1	1.8 ± 0.1	1.8 ± 0.1	1.8 ± 0.1
Weight (kg)	69.2 ± 11.1	71.8 ± 10.8	70.1 ± 11.7	70.2 ± 11.4
Body mass index (kg/m^2^)	21.5 ± 2.4	22.1 ± 2.2	21.5 ± 2.4	21.7 ± 2.4
Body surface area (m^2^)	1.9 ± 0.2	1.9 ± 0.2	1.9 ± 0.2	1.9 ± 0.2
Heart rate	68.6 ± 12.9	63.8 ± 10.1	69.1 ± 9	63.6 ± 9.7
Systolic blood pressure (mmHg)	123.2 ± 12.4	120.2 ± 7.4	123.1 ± 9.7	118.5 ± 11.5
Diastolic blood pressure (mmHg)	69.7 ± 14.4	66.9 ± 11.6	77.5 ± 9.5	68.6 ± 7.9

Mean ± standard deviation.

Heart rate and blood pressure were measured automatically oscillometric during the positioning of the patient in each scanner.

### 3.2 Inter-site comparability

**Figure 3:**
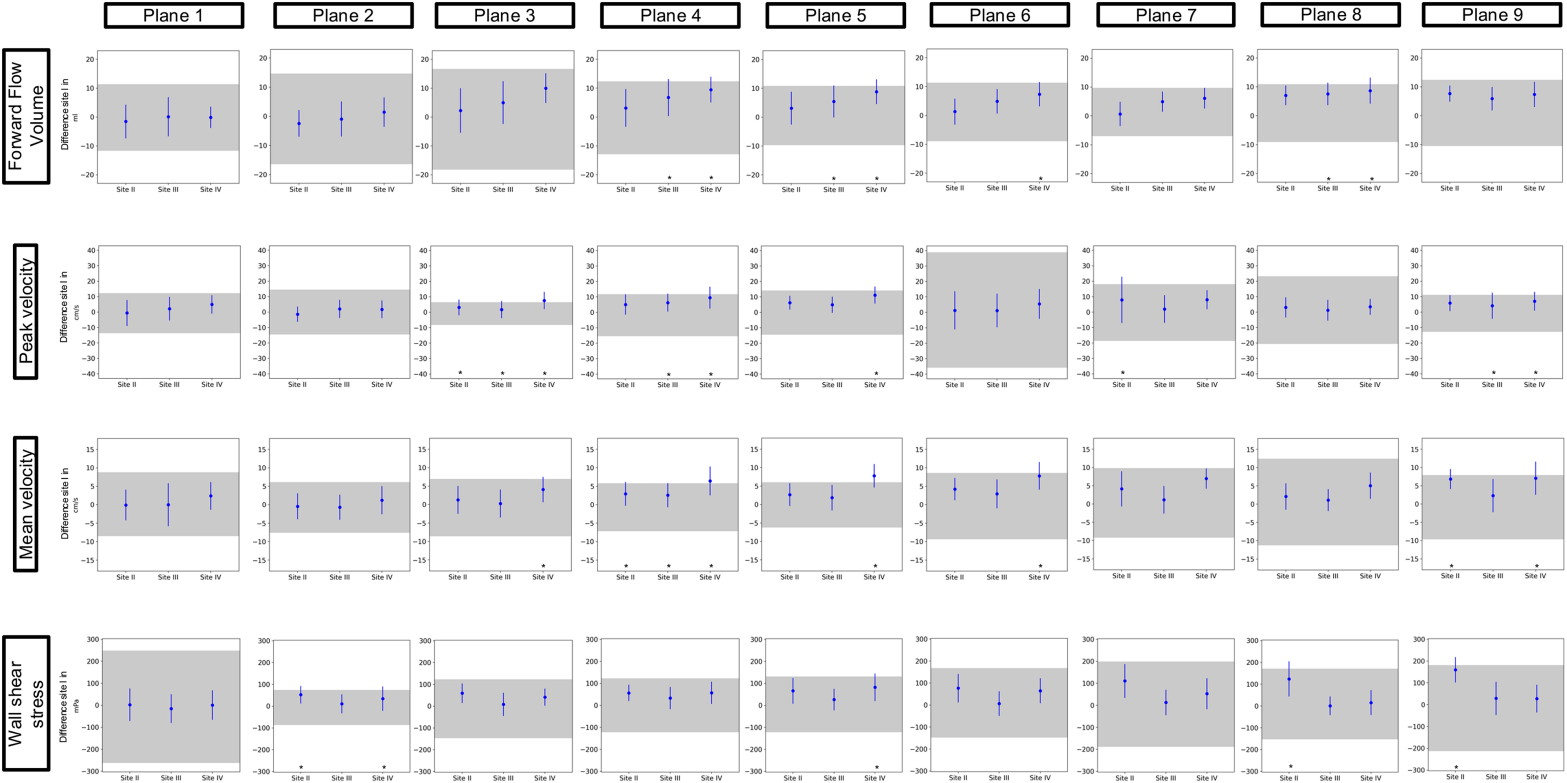
Inter-site comparison for the 4D flow CMR aorta protocol. The grayish area represents the means and 95% tolerance ranges of both scans before and after repositioning at study Site I serving as a reference for the site comparison. The results of the individual sites are represented by the mean deviation with a 95% confidence interval (blue lines with dots). If these were within the tolerance ranges equivalence was assumed. Exceeded tolerance ranges are indicated by *.

**Figure 4:**
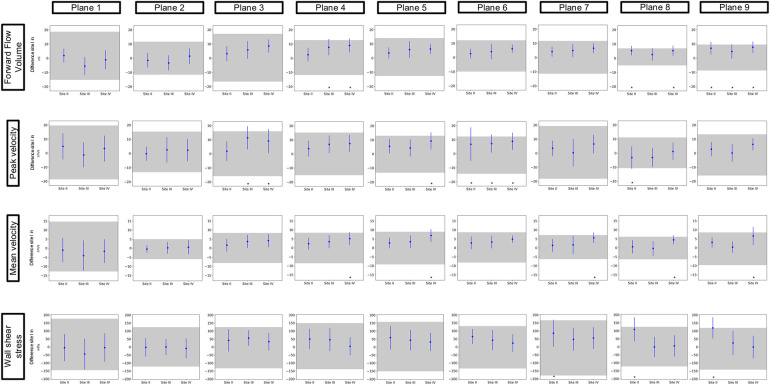
Inter-site comparison for the 4D flow CMR whole heart protocol. The grayish area represents the means and 95% tolerance ranges of both scans before and after repositioning at study Site I serving as a reference for the site comparison. The results of the individual sites are represented by the mean deviation with a 95% confidence interval (blue lines with dots). If these were within the tolerance ranges equivalence was assumed. Exceeded tolerance ranges are indicated by *.

Figures 3 and 4 show the results of the inter-site comparability for the aorta and whole heart protocol, respectively. In P1 and P2, FFV, Vm, and Vp were within the defined TOL at all study sites. In four planes for FFV, five for Vm, and five for Vp, TOL were exceeded in the aorta protocol, compared to three planes for FFV, five for Vm, and four for Vp in the whole heart protocol. 3D WSS shows exceeded TOL for four planes in the aorta protocol, including P2, compared to three planes in the whole heart protocol. Overall, Site II exceeded TOL in 7 planes, Site III in 7 planes, and Site IV in 15 planes of the aorta protocol, compared to 7 planes, 4 planes, and 11 planes of the whole heart protocol.

Mean values ± standard deviation per plane for all parameters analyzed are displayed in Tables 3 and 4 for the aorta and whole heart protocol, respectively.

**Table 3:** Mean values ± standard deviation of forward flow volume, mean and peak velocity, and 3D WSS in the aorta protocol.

Parameter	Forward flow volume (mL)	Peak velocity (cm/s)	Mean velocity (cm/s)	3D wall shear stress (mPa)
Plane 1	98.61 ± 18.55	121.44 ± 15.56	85.30 ± 12.16	1,014.47 ± 161.15
Plane 2	89.21 ± 19.18	93.73 ± 14.01	60.69 ± 12.16	675.25 ± 107.73
Plane 3	85.90 ± 19.99	94.25 ± 15.58	62.72 ± 10.22	755.76 ± 106.87
Plane 4	69.55 ± 15.94	93.18 ± 14.35	60.33 ± 9.62	751.97 ± 127.69
Plane 5	62.53 ± 15.23	93.49 ± 13.40	62.64 ± 9.00	847.33 ± 134.18
Plane 6	56.96 ± 13.78	96.26 ± 14.76	64.22 ± 8.72	888.52 ± 127.27
Plane 7	56.77 ± 13.80	108.46 ± 19.09	71.58 ± 10.45	1,002.15 ± 142.88
Plane 8	58.22 ± 13.81	108.36 ± 16.97	72.00 ± 10.97	995.24 ± 157.15
Plane 9	58.97 ± 13.46	105.87 ± 15.44	74.20 ± 10.55	1,055.18 ± 167.64

Mean ± standard deviation.

**Table 4:** Mean values ± standard deviation of forward flow volume, mean and peak velocity, and 3D WSS in the whole heart protocol.

Parameter	Forward flow volume (mL)	Peak velocity (cm/s)	Mean velocity (cm/s)	3D wall shear stress (mPa)
Plane 1	97.31 ± 21.43	121.96 ± 17.09	80.87 ± 11.48	1,006.18 ± 176.28
Plane 2	86.55 ± 18.80	92.36 ± 17.17	58.94 ± 11.61	678.68 ± 113.83
Plane 3	85.86 ± 19.30	93.86 ± 18.90	61.13 ± 10.50	782.13 ± 126.68
Plane 4	70.93 ± 17.04	90.69 ± 14.10	58.50 ± 9.81	778.14 ± 140.78
Plane 5	64.57 ± 16.25	94.04 ± 13.69	62.44 ± 8.95	877.73 ± 136.25
Plane 6	58.21 ± 14.69	96.76 ± 15.13	63.78 ± 8.86	907.69 ± 124.39
Plane 7	57.21 ± 14.78	104.19 ± 16.04	69.49 ± 10.83	989.79 ± 141.75
Plane 8	59.34 ± 13.55	106.39 ± 15.79	70.53 ± 9.59	986.18 ± 161.30
Plane 9	57.73 ± 12.81	104.60 ± 13.94	73.55 ± 10.92	1,025.11 ± 169.59

Mean ± standard deviation.

**Figure 5:**
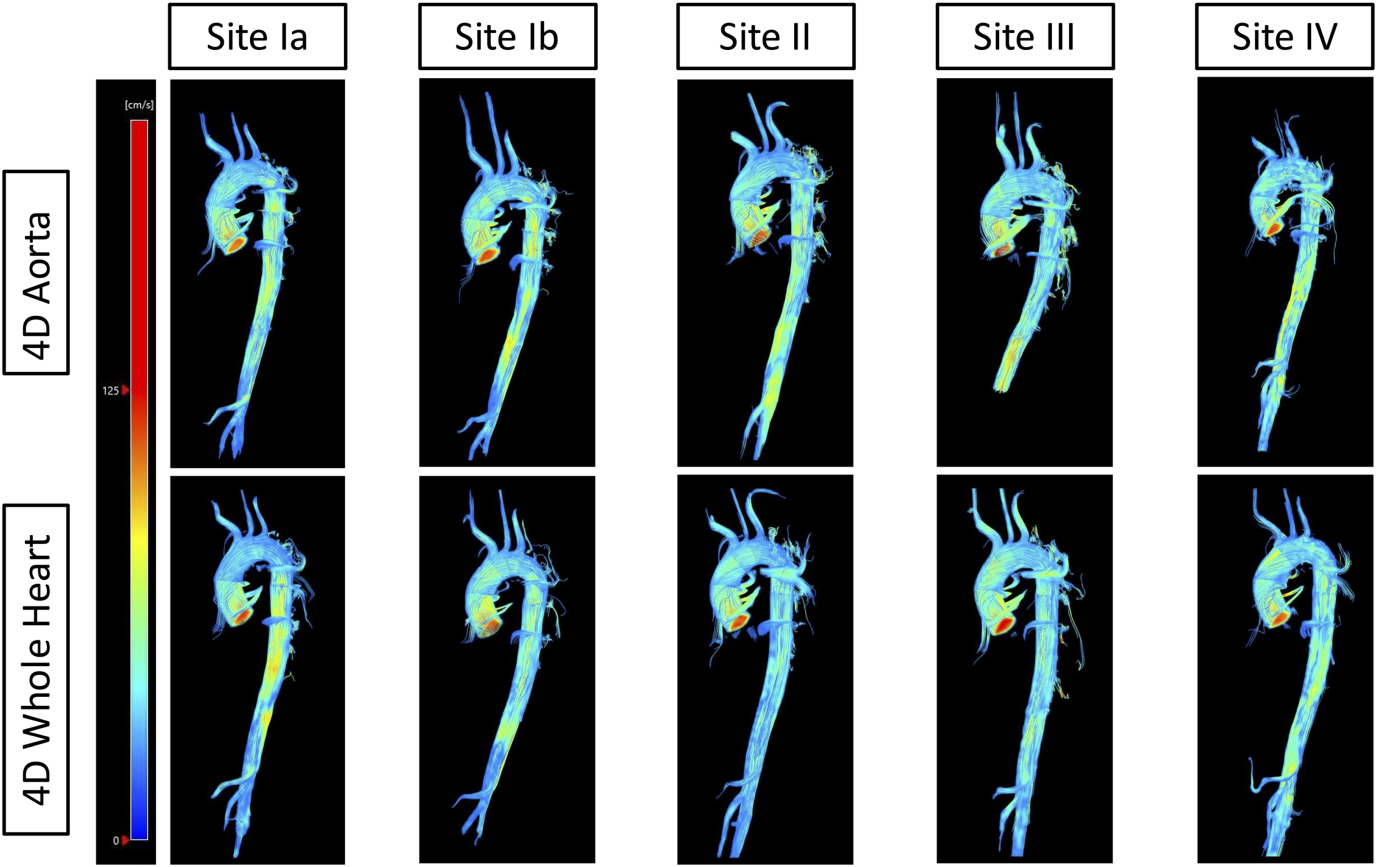
Streamlines of one traveling volunteer across protocols and sites. The top row displays streamlines of the 4D flow CMR aorta protocol of one traveling volunteer across all sites. The bottom row displays streamlines of the 4D flow CMR whole heart protocol of the same traveling volunteer across all sites.

Figure 5 displays the visual results of both protocols at each site for one traveling volunteer. The Supplementary Material contains pathline videos of one traveling volunteer of both protocols at each site.

### 3.3 Protocol comparability

Table 5 displays the mean ± 95% limits of agreement and ICC values with their 95% CI of the protocol comparability. ICC showed excellent agreement for FFV and good to excellent agreement for Vm and 3D WSS in every plane. Vp showed good to excellent agreement in eight planes and moderate agreement in one plane (P6).

**Table 5:** Differences between the two protocols (aorta and whole heart) for forward flow volume, mean and peak velocity, and 3D WSS.

Parameter	Difference of forward flow volume (mL) |ICC|	Difference of peak velocity (cm/s) |ICC|	Difference of mean velocity (cm/s) |ICC|	Difference of 3D wall shear stress (mPa) |ICC|
Plane 1	1.8 ± 8.9 |0.95| (0.92–0.97)	−0.1 ± 10.7 |0.88| (0.82–0.93)	4.5 ± 9.3 |0.79| (0.61–0.88)	14.3 ± 113.7 |0.87| (0.81–0.92)
Plane 2	3.0 ± 6.0 |0.97| (0.93–0.98)	1.0 ± 10.6 |0.87| (0.80–0.92)	1.6 ± 4.5 |0.96| (0.93–0.98)	−1.3 ± 62.9 |0.91| (0.87–0.94)
Plane 3	0.4 ± 5.9 |0.98| (0.97–0.99)	0.5 ± 11.6 |0.88| (0.81–0.92)	1.7 ± 3.5 |0.97| (0.93–0.98)	−23.0 ± 77.1 |0.87| (0.79–0.92)
Plane 4	−1.2 ± 5.6 |0.97| (0.95–0.98)	2.8 ± 7.2 |0.92| (0.87–0.96)	2.0 ± 3.3 |0.96| (0.90–0.98)	−26.0 ± 76.5 |0.91| (0.84–0.94)
Plane 5	−1.7 ± 4.5 |0.98| (0.96–0.99)	−0.6 ± 7.5 |0.92| (0.88–0.95)	0.3 ± 3.4 |0.96| (0.94–0.98)	−33.2 ± 65.9 |0.92| (0.84–0.96)
Plane 6	−0.9 ± 4.5 |0.98| (0.96–0.98)	−0.4 ± 15.0 |0.67| (0.49–0.79)	0.6 ± 4.9 |0.92| (0.87–0.95)	−15.6 ± 76.9 |0.89| (0.83–0.93)
Plane 7	−0.4 ± 4.5 |0.98| (0.96–0.98)	4.6 ± 14.3 |0.80| (0.67–0.87)	2.4 ± 5.2 |0.93| (0.86–0.96)	16.4 ± 92.0 |0.88| (0.82–0.93)
Plane 8	−1.0 ± 4.5 |0.97| (0.96–0.98)	2.5 ± 8.6 |0.92| (0.88–0.95)	1.8 ± 5.0 |0.93| (0.89–0.96)	15.8 ± 81.3 |0.93| (0.89–0.95)
Plane 9	1.3 ± 5.0 |0.96| (0.94–0.98)	1.7 ± 9.8 |0.88| (0.81–0.92)	0.8 ± 6.9 |0.88| (0.82–0.93)	35.5 ± 79.8 |0.93| (0.87–0.96)

Mean ± standard deviation.

|ICC| (CI).

**Figure 6:**
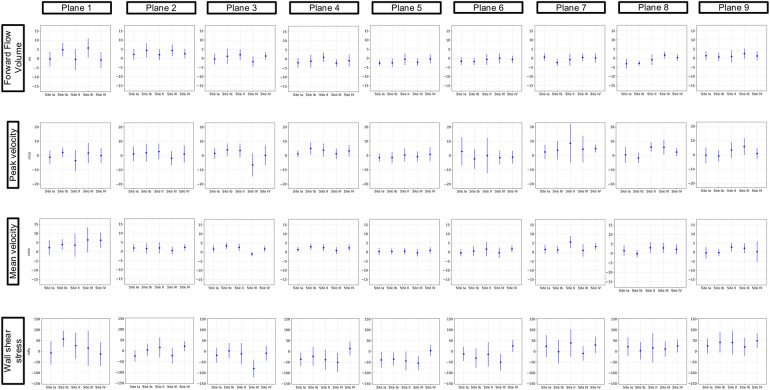
Comparison of the aorta and whole heart protocol. The blue dot represents a mean value based on the corresponding values of both scans. The blue lines show the mean deviations of the two scans from that mean value.

Figure 6 displays the results of the protocol comparison depicting a mean value based on the corresponding values of both scans (blue dot) as well as the mean deviation (blue line). At Site II in P7, Vp presented a mean deviation of up to 0.22 m/s between the aorta and whole heart protocol. Vm showed fewer differences between the two protocols than Vp in P2–P9. P1 at Site III revealed a wider mean deviation in Vm than in Vp (0.13 > 0.08 m/s). Vp at Site II showed a mean deviation of up to 0.11 m/s in P1. FFV showed a mean deviation of up to 11 mL in P1 at Site I. Sites II–IV presented <9 mL difference between the two protocols in every plane. 3D WSS showed differences of up to 121 mPa in P3 at Site III between the aorta and whole heart protocol.

### 3.4 Inter- and intra-observer

**Figure 7:**
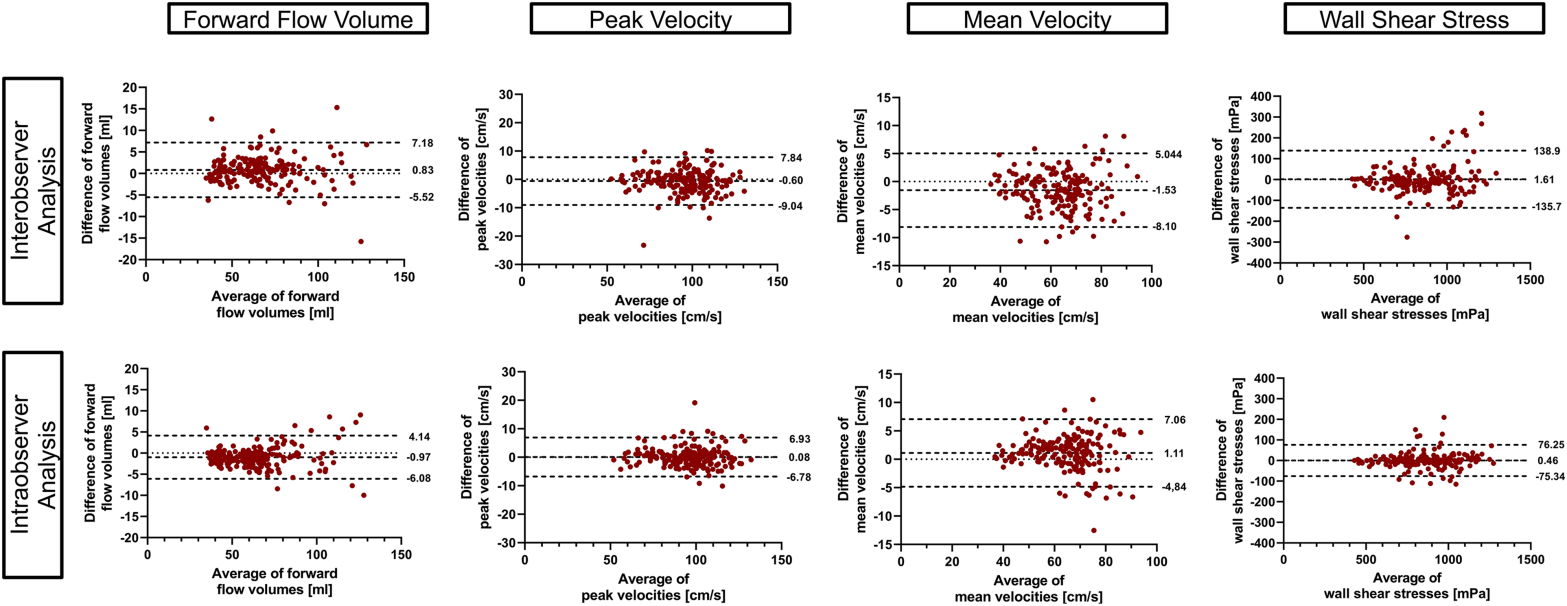
Inter- and intra-observer comparison. Bland–Altman plots using 95% tolerance intervals for inter- and intra-observer comparison have been generated using the aorta and the whole heart protocol. The three dashed lines represent the mean difference ± 95% tolerance interval for each parameter.

Bland–Altmann plots for inter- and intra-observer analysis are displayed in Figure 7 and revealed good to excellent agreement. ICC values showed excellent agreement for inter-observer analysis [FFV = 0.99 (0.99–1.0), Vm = 0.98 (0.96–0.99), Vp = 0.98 (0.98–0.99), and 3D WSS = 0.97 (0.95–0.97)] as well as intra-observer analysis [FFV = 0.99 (0.99–1.0), Vm = 0.98 (0.97–0.99), Vp = 0.99 (0.98–0.99), and 3D WSS = 0.99 (0.99–0.99)].

The average time span between the first and second analysis of the intra-observer analysis was 79.4 ± 14.5 days.

## 4 Discussion

The main findings of our study are as follows: At the sinotubular junction and the ascending aorta, different scanners from the same vendor with the same field strength and different field strengths show excellent agreement for flow parameters. However, different field strength leads to more disagreement in other regions of the thoracic aorta. 3D WSS shows relatively good agreement as well; however, wide TOL for 3D WSS have to be considered when analyzing the agreement between different sites. The comparison of the 4D flow CMR aorta and 4D flow CMR whole heart protocol showed a good agreement.

Inter-site comparison of flow parameters did not show any disagreement in both protocols in P1 and P2. However, it has to be said that these planes also have wider TOL than some other planes of the respective value. Demir et al. investigated the inter-vendor comparability of 4D flow CMR-derived hemodynamic parameters. Their study revealed that despite the same field strength hemodynamic parameters show significant differences when derived from scanners of different MRI vendors. Following their findings, they stated that, similar to our findings, the sinotubular junction was considered the most stable plane location with the least significant differences (13). Especially P1 plays an important role in clinical routine as 2D flow measurements of flow in the ascending aorta take place in this plane (23).

Punzo et al. reported that different field strengths result in significant differences in flow parameters. In their study, they described significant differences in flow parameters between different scanners from the same vendor (1.5 T and 3 T) and a scanner from another vendor (3 T). Similar to their findings, our results show inequivalence in P3–P9 (24). The authors assumed that the discovered differences might relate to the different vendors. Another possible explanation for disagreements in P3–P9 might be a higher distance from the isocenter, as it has been shown that flow analysis in regions further away from the isocenter results in less accurate results (13, 25). Another possible explanation is the branching vessels and thus slightly altered flow patterns in the aortic arch. As a consequence, small alterations in plane positioning in this region of the thoracic aorta might lead to significant changes in the parameters obtained from 4D flow imaging. However, this explanation does not account for surpassed TOL in the descending aorta.

In addition to the influence of scanner configurations and the protocol in use, a further aspect one has to consider when assessing the reproducibility of 4D flow CMR are subject-dependent parameters, e.g., heart rate and blood pressure (2). It is known that elevated mean pulmonary arterial pressure can lead to a specific vortical blood flow along the main pulmonary artery (26). Furthermore, body size and heart rate can influence temporal and spatial resolution (2). These aspects have to be taken into consideration when interpreting the results of our study due to the inclusion criteria of our study and thus healthy cohort. Needless to say, patients might present with the above-mentioned alterations and illnesses which might affect the reproducibility of 4D flow CMR measurements.

Inter-site comparison of 3D WSS exceeded TOL in a similar number of planes as flow parameters but also in P2. It has to be made clear that when interpreting the results of this study, TOL for 3D WSS parameters show a wide span with regard to the absolute values. It is known that WSS parameters are highly sensitive to small changes in multiple parameters such as temporal and spatial resolution, plane positioning, and vessel contours in postprocessing, due to the way they are calculated (15, 27). This can lead to larger differences in the values generated.

Van der Palen et al. investigated the reproducibility of segmental aortic WSS. They were able to show that WSS does show more reliability in the ascending aorta than in the aortic arch and the descending aorta (28). In accordance with their findings, our results show good agreement in the ascending aorta as well, except for P2 of the aorta protocol at Sites II and IV. It has to be highlighted that in this plane of the aorta protocol, TOL are narrower than in all the other planes of both protocols. Exceeded TOL therefore could be a possible consequence of this circumstance. It can only be assumed that this plane shows smaller tolerance ranges due to the absence of branching vessels or the aortic valve in close proximity, which as mentioned above could possibly influence flow patterns, thus resulting in larger differences between sites and larger TOL. However, to make 3D WSS a reliable parameter in clinical routine, one should aim for smaller TOL. One possible approach to achieve said reduction of TOL could be using volumes instead of planes when analyzing 3D WSS. Due to the larger number of values included in the calculation of 3D WSS, it might be less sensitive to changes in the vessel wall.

The comparison of the aorta and whole heart protocols showed excellent to good agreement for flow parameters except for P6. Vp showed a mean deviation of up to 0.11 m/s and a forward flow volume of up to 11 mL at the sinotubular junction and in the ascending aorta. It is known that basic hemodynamic parameters such as blood flow velocity and stroke volume show significant changes even in healthy volunteers. Traber et al. demonstrated the differences in stroke volume from beat to beat even in sinus rhythm with a variation of up to 33% (29). In their study, they cited physiological heartbeat variability and different breath-holding levels as possible explanations for these results. Considering the multitude of factors affecting stroke volume, e.g., an increase in peak force generated during the contraction and the Frank–Starling mechanism, we considered these results reliable (30). Furthermore, they were able to show that Vp in the ascending aorta was 121 ± 24.0 cm/s in a group of 51 healthy volunteers (29).

Currently in clinical routine, evaluation of the left ventricular ejection fraction (LVEF) and thus stroke volume is often based on transthoracic echocardiography. Literature states that differences in LVEF of up to 10% do not necessarily represent an actual change in systolic function between two measurements. This is rather a consequence of inconsistent image quality and different loading conditions of the patient (31).

Following these findings and with regard to the clinical application of FFV and Vp in the ascending aorta and the sinotubular junction the differences between the two protocols have been interpreted as acceptable.

In accordance with inter-site comparison, 3D WSS shows relatively high differences between the two protocols in the aortic arch and descending thoracic aorta, with regard to the absolute values. Note, however, that P1 shows similar differences as P3–P9. However, ICC values indicate good to excellent agreement between the two protocols in every plane.

To the best of our knowledge, this is the first study comparing two protocols of a 4D flow CMR research sequence acquiring flow information in the thoracic aorta solely and the thoracic aorta and whole heart combined. Wiesemann et al. compared different sequences for 4D flow CMR image acquisition (14). In their study, they used three sequences comparing basic and advanced hemodynamic parameters at a 1.5 T scanner. Their results display significant differences in WSS between the sequences in use. Our findings do not concur with this since ICC values show good to excellent agreement for the protocols in use. 3D WSS was therefore interpreted as comparable between the two protocols.

The results of this study show that 4D flow CMR shows similar results in a highly controlled environment of scanners of the same manufacturer and field strength. However, technical changes, such as a different field strength lead to relevant uncertainties. Following these findings in the future, it is important to further investigate other possible confounders, i.e., distance from the isocenter to make 4D flow CMR reproducible not only in the ascending aorta but the entire thoracic aorta. Moreover, it is important to assess whether 4D flow CMR is not only reproducible but shows equivalent results to 2D flow CMR and therefore can be considered clinically reliable. However, before actually including 4D flow CMR in clinical routine, it is indispensable to generate clinically applicable reference values as it has been shown that age differences and sex lead to significant changes in basic and advanced hemodynamic parameters derived from 4D flow CMR (32).

### 4.1 Limitations

The main limitation of the study is the small number of healthy traveling volunteers enrolled as a consequence of organizational challenges such as the traveling effort. However, each traveling volunteer enrolled received a total of five respectively four scans with two 4D flow CMR protocols. Therefore, we firmly believe that the total amount of measurements included in this study constitutes a sufficiently large sample size to perform an adequate statistical analysis.

Due to the traveling effort as well as limited access to the scanners during COVID-19, traveling volunteers could not be scanned at all sites within one day. As a consequence, physiological variations and changes over time might have affected the results of this study.

The mean age of the study cohort is relatively young, reducing application to clinical scenarios.

Due to postprocessing issues and insufficient image quality three aorta and six whole heart scans had to be excluded from the statistical analysis.

A further limitation of our study is the usage of planes when analyzing 3D WSS along the thoracic aorta. As mentioned above, when using volumes instead of planes, a larger number of values is included in the calculation of 3D WSS within these volumes. Therefore, single values have less influence and a better agreement between the sites might be achieved.

## 5 Conclusion

4D flow CMR shows a good agreement between different scanners from the same vendor with the same field strength in clinically relevant areas for flow parameters. The aorta and whole heart show good agreement in flow parameters.

## Data Availability

The datasets for this study are not readily available due to German data protection laws. Requests to access these datasets should be directed to jeanette.schulz-menger@charite.de.

## Supplementary material

Supplementary material (Each video visualizes the results of the same traveling volunteer)

Supplementary Video 1 Site Ia aorta: Pathlines of one traveling volunteer of the aorta protocol of the first scan at Site I.

Supplementary Video 2 Site Ib aorta: Pathlines of one traveling volunteer of the aorta protocol of the second scan at Site I.

Supplementary Video 3 Site II aorta: Pathlines of one traveling volunteer of the aorta protocol of the scan at Site II.

Supplementary Video 4 Site III aorta: Pathlines of one traveling volunteer of the aorta protocol of the scan at Site III.

Supplementary Video 5 Site IV aorta: Pathlines of one traveling volunteer of the aorta protocol of the scan at Site IV.

Supplementary Video 6 Site Ia whole heart: Pathlines of one traveling volunteer of the whole heart protocol of the first scan at Site I.

Supplementary Video 7 Site Ib whole heart: Pathlines of one traveling volunteer of the whole heart protocol of the second scan at Site I.

Supplementary Video 8 Site II whole heart: Pathlines of one traveling volunteer of the whole heart protocol of the scan at Site II.

Supplementary Video 9 Site III whole heart: Pathlines of one traveling volunteer of the whole heart protocol of the scan at Site III.

Supplementary Video 10 Site IV whole heart: Pathlines of one traveling volunteer of the whole heart protocol of the scan at Site IV.

## References

[1] Paddock S Tsampasian V Assadi H Calife Mota B Swift AJ Chowdhary A Clinical translation of three-dimensional scar, diffusion tensor imaging, four-dimensional flow, and quantitative perfusion in cardiac MRI: a comprehensive review. Front Cardiovasc Med. (2021) 8:682027. 10.3389/fcvm.2021.682027 34307496 PMC8292630

[2] Bissell MM Raimondi F Ait Ali L Allen BD Barker AJ Bolger A 4D flow cardiovascular magnetic resonance consensus statement: 2023 update. J Cardiovasc Magn Reson. (2023) 25(1):40. 10.1186/s12968-023-00942-z 37474977 PMC10357639

[3] Geeraert P Jamalidinan F Fatehi Hassanabad A Sojoudi A Bristow M Lydell C Bicuspid aortic valve disease is associated with abnormal wall shear stress, viscous energy loss, and pressure drop within the ascending thoracic aorta: a cross-sectional study. Medicine (Baltimore). (2021) 100(26):e26518. 10.1097/MD.0000000000026518 34190185 PMC8257908

[4] Bürk J Blanke P Stankovic Z Barker A Russe M Geiger J Evaluation of 3D blood flow patterns and wall shear stress in the normal and dilated thoracic aorta using flow-sensitive 4D CMR. J Cardiovasc Magn Reson. (2012) 14(1):84. 10.1186/1532-429X-14-84 23237187 PMC3534249

[5] Bissell MM Hess AT Biasiolli L Glaze SJ Loudon M Pitcher A Aortic dilation in bicuspid aortic valve disease: flow pattern is a major contributor and differs with valve fusion type. Circ Cardiovasc Imaging. (2013) 6(4):499–507. 10.1161/CIRCIMAGING.113.000528 23771987 PMC3859916

[6] Soulat G Scott MB Allen BD Avery R Bonow RO Malaisrie SC Association of regional wall shear stress and progressive ascending aorta dilation in bicuspid aortic valve. JACC Cardiovasc Imaging. (2022) 15(1):33–42. 10.1016/j.jcmg.2021.06.020 34419402 PMC8741630

[7] Minderhoud SCS Roos-Hesselink JW Chelu RG Bons LR van den Hoven AT Korteland SA Wall shear stress angle is associated with aortic growth in bicuspid aortic valve patients. Eur Heart J Cardiovasc Imaging. (2022) 17(23):1680–9. 10.1093/ehjci/jeab290 PMC9671285 34977931

[8] Guala A Dux-Santoy L Teixido-Tura G Ruiz-Muñoz A Galian-Gay L Luz Servato M Wall shear stress predicts aortic dilation in patients with bicuspid aortic valve. JACC Cardiovasc Imaging. (2022) 15(1):46–56. 10.1016/j.jcmg.2021.09.023 34801463

[9] Kilner PJ Gatehouse PD Firmin DN. Flow measurement by magnetic resonance: a unique asset worth optimising. J Cardiovasc Magn Reson. (2007) 9(4):723–8. 10.1080/10976640701465090 17613655

[10] Biglands JD Radjenovic A Ridgway JP. Cardiovascular magnetic resonance physics for clinicians: part II. J Cardiovasc Magn Reson. (2012) 14(1):66. 10.1186/1532-429X-14-66 22995744 PMC3533879

[11] Safarkhanlo Y Jung B Bernhard B Peper ES Kwong RY Bastiaansen JAM Mitral valve regurgitation assessed by intraventricular CMR 4D-flow: a systematic review on the technological aspects and potential clinical applications. Int J Cardiovasc Imaging. (2023) 39:1963–77. 10.1007/s10554-023-02893-z 37322317 PMC10589148

[12] Vasanawala SS Hanneman K Alley MT Hsiao A. Congenital heart disease assessment with 4D flow MRI. J Magn Reson Imaging. (2015) 42(4):870–86. 10.1002/jmri.24856 25708923

[13] Demir A Wiesemann S Erley J Schmitter S Trauzeddel RF Pieske B Traveling volunteers: a multi-vendor, multi-center study on reproducibility and comparability of 4D flow derived aortic hemodynamics in cardiovascular magnetic resonance. J Magn Reson Imaging. (2022) 55(1):211–22. 10.1002/jmri.27804 34173297

[14] Wiesemann S Schmitter S Demir A Prothmann M Schwenke C Chawla A Impact of sequence type and field strength (1.5, 3, and 7T) on 4D flow MRI hemodynamic aortic parameters in healthy volunteers. Magn Reson Imaging. (2021) 85(2):721–33. 10.1002/mrm.28450 32754969

[15] Oechtering TH Nowak A Sieren MM Stroth AM Kirschke N Wegner F Repeatability and reproducibility of various 4D flow MRI postprocessing software programs in a multi-software and multi-vendor cross-over comparison study. J Cardiovasc Magn Reson. (2023) 25(1):22. 10.1186/s12968-023-00921-4 36978131 PMC10052852

[16] Schmidt S Flassbeck S Schmelter S Schmeyer E Ladd ME Schmitter S. The impact of 4D flow displacement artifacts on wall shear stress estimation. Magn Reson Med. (2021) 85(6):3154–68. 10.1002/mrm.28641 33421221

[17] Groschel J Trauzeddel RF Muller M von Knobelsdorff-Brenkenhoff F Viezzer D Hadler T Multi-site comparison of parametric T1 and T2 mapping: healthy travelling volunteers in the Berlin research network for cardiovascular magnetic resonance (BER-CMR). J Cardiovasc Magn Reson. (2023) 25(1):47. 10.1186/s12968-023-00954-9 37574535 PMC10424349

[18] Kramer CM Barkhausen J Bucciarelli-Ducci C Flamm SD Kim RJ Nagel E. Standardized cardiovascular magnetic resonance imaging (CMR) protocols: 2020 update. J Cardiovasc Magn Reson. (2020) 22(1):17. 10.1186/s12968-020-00607-1 32089132 PMC7038611

[19] Jacobs K Hahn L Horowitz M Kligerman S Vasanawala S Hsiao A. Hemodynamic assessment of structural heart disease using 4D flow MRI: how we do it. AJR Am J Roentgenol. (2021) 217(6):1322–32. 10.2214/AJR.21.25978 34076463

[20] Ramaekers M Westenberg JJM Adriaans BP Nijssen EC Wildberger JE Lamb HJ A clinician’s guide to understanding aortic 4D flow MRI. Insights Imaging. (2023) 14(1):114. 10.1186/s13244-023-01458-x 37395817 PMC10317921

[21] Zange L Muehlberg F Blaszczyk E Schwenke S Traber J Funk S Quantification in cardiovascular magnetic resonance: agreement of software from three different vendors on assessment of left ventricular function, 2D flow and parametric mapping. J Cardiovasc Magn Reson. (2019) 21(1):12. 10.1186/s12968-019-0522-y 30786898 PMC6383230

[22] Koo TK Li MY. A guideline of selecting and reporting intraclass correlation coefficients for reliability research. J Chiropr Med. (2016) 15(2):155–63. 10.1016/j.jcm.2016.02.012 27330520 PMC4913118

[23] Gabbour M Schnell S Jarvis K Robinson JD Markl M Rigsby CK. 4-D flow magnetic resonance imaging: blood flow quantification compared to 2-D phase-contrast magnetic resonance imaging and Doppler echocardiography. Pediatr Radiol. (2015) 45(6):804–13. 10.1007/s00247-014-3246-z 25487721 PMC4450116

[24] Punzo B Ranieri B Tramontano L Affinito O Franzese M Bossone E 4D-flow cardiovascular magnetic resonance sequence for aortic assessment: multi-vendor and multi-magnetic field reproducibility in healthy volunteers. J Clin Med. (2023) 12(8):2960. 10.3390/jcm12082960 37109295 PMC10141060

[25] Gatehouse PD Rolf MP Graves MJ Hofman MB Totman J Werner B Flow measurement by cardiovascular magnetic resonance: a multi-centre multi-vendor study of background phase offset errors that can compromise the accuracy of derived regurgitant or shunt flow measurements. J Cardiovasc Magn Reson. (2010) 12(1):5. 10.1186/1532-429X-12-5 20074359 PMC2818657

[26] Krauter C Reiter U Kovacs G Reiter C Masana M Olschewski H Automated vortical blood flow-based estimation of mean pulmonary arterial pressure from 4D flow MRI. Magn Reson Imaging. (2022) 88:132–41. 10.1016/j.mri.2022.02.007 35189283

[27] Petersson S Dyverfeldt P Ebbers T. Assessment of the accuracy of MRI wall shear stress estimation using numerical simulations. J Magn Reson Imaging. (2012) 36(1):128–38. 10.1002/jmri.23610 22336966

[28] van der Palen RLF Roest AAW van den Boogaard PJ de Roos A Blom NA Westenberg JJM. Scan-rescan reproducibility of segmental aortic wall shear stress as assessed by phase-specific segmentation with 4D flow MRI in healthy volunteers. Magma. (2018) 31(5):653–63. 10.1007/s10334-018-0688-6 29804208 PMC6132557

[29] Traber J Wurche L Dieringer MA Utz W von Knobelsdorff-Brenkenhoff F Greiser A Real-time phase contrast magnetic resonance imaging for assessment of haemodynamics: from phantom to patients. Eur Radiol. (2016) 26(4):986–96. 10.1007/s00330-015-3897-7 26188655

[30] Sequeira V van der Velden J. Historical perspective on heart function: the Frank-Starling law. Biophys Rev. (2015) 7(4):421–47. 10.1007/s12551-015-0184-4 28510104 PMC5418489

[31] Klaeboe LG Edvardsen T. Echocardiographic assessment of left ventricular systolic function. J Echocardiogr. (2019) 17(1):10–6. 10.1007/s12574-018-0405-5 30390189

[32] Schafstedde M Jarmatz L Bruning J Hullebrand M Nordmeyer S Harloff A Population-based reference values for 4D flow MRI derived aortic blood flow parameters. Physiol Meas. (2023) 44(3):035003. 10.1088/1361-6579/acb8fd 36735968

